# Comment on Savioli et al. Mild Head Trauma: Is Antiplatelet Therapy a Risk Factor for Hemorrhagic Complications? *Medicina* 2021, *57*, 357

**DOI:** 10.3390/medicina57090887

**Published:** 2021-08-27

**Authors:** Bartolomeo Lorenzati, Alice Bruno, Giulia Racca, Jacopo Giamello, Attilio Allione, Letizia Barutta, Emanuele Bernardi, Ilaria Blangetti, Alessia Bono, Luca Dutto, Chiara Fulcheri, Remo Galaverna, Alberto Grosso, Elena Maggio, Gianpiero Martini, Vincenzo Peloponneso, Massimo Perotto, Tania Prinzis, Paola Ramonda, Alessandro Raviolo, Massimo Rega, Andrea Tortore, Francesco Tosello, Andrea Sciolla, Giuseppe Lauria, on behalf of “Trauma Management Course” Faculty

**Affiliations:** 1 Emergency Medicine, Department of Emergency and Critical Care, A.O. S. Croce e Carle Hospital, 12100 Cuneo, Italy; allione.a@ospedale.cuneo.it (A.A.); barutta.l@ospedale.cuneo.it (L.B.); bernardi.e@ospedale.cuneo.it (E.B.); bono.a@ospedale.cuneo.it (A.B.); dutto.l@ospedale.cuneo.it (L.D.); fulcheri.c@ospedale.cuneo.it (C.F.); grosso.a@ospedale.cuneo.it (A.G.); maggio.e@ospedale.cuneo.it (E.M.); martini.g@ospedale.cuneo.it (G.M.); peloponneso.v@ospedale.cuneo.it (V.P.); prinzis.t@ospedale.cuneo.it (T.P.); raviolo.a@ospedale.cuneo.it (A.R.); rega.m@ospedale.cuneo.it (M.R.); tortore.a@ospedale.cuneo.it (A.T.); tosello.f@ospedale.cuneo.it (F.T.); sciolla.a@ospedale.cuneo.it (A.S.); lauria.g@ospedale.cuneo.it (G.L.); 2 School of Emergency Medicine, University of Pavia, 27100 Pavia, Italy; dutto.a@ospedale.cuneo.it; 3 Internal Medicine, Department of Medicine, A.O. S. Croce e Carle Hospital, 12100 Cuneo, Italy; racca.g@ospedale.cuneo.it; 4 School of Emergency Medicine, University of Turin, 10100 Turin, Italy; giamello.j@ospedale.cuneo.it; 5 Intensive Care Unit, Regina Montis Regalis Hospital, 12084 Mondovi, Italy; blangetti.i@ospedale.cuneo.it; 6 Intensive Care Unit, Department of Emergency and Critical Care, A.O. S. Croce e Carle Hospital, 12100 Cuneo, Italy; galaverna.r@ospedale.cuneo.it (R.G.); ramonda.p@ospedale.cuneo.it (P.R.); 7 Internal Medicine, Department of Medicine, P.O. Michele Ferrero Hospital, 12060 Verduno, Italy; perotto.m@ospedale.cuneo.it

We read the article of Savioli G. et al. with interest, titled Mild Head Trauma: Is Antiplatelet Therapy a Risk Factor for Hemorrhagic Complications? [1], which was recently published in your journal.

We appreciate this contribution because minor head trauma (MHT) is common in the Emergency Department (ED) with an average incidence in Europe of 232 cases per 100,000 inhabitants. However, its management is controversial and irregular, especially in patients treated with the vitamin K antagonist (VKA), new oral direct anticoagulants (DOACs), and antiplatelet agents (APT). In these patients, international guidelines recommend a head computed tomography (HCT) as a first evaluation on arrival in ED regardless of the patient’s clinical presentation, followed by a second HCT 24–48 h later despite clinical and neurological stable conditions. Furthermore, the literature contains much debate as to whether APT predisposes patients to bleeding in the event of mild head trauma. A few years ago, we planned a study on patients in therapy with oral anticoagulant (VKA or DOACs) or antiplatelets (APT) who were firstly evaluated in our ED and then observed in the Observation Unit for minor head trauma. We recorded the incidence of intracranial hemorrhaging (ICH) during the first and second HCT, and finally we tried to identify clinical predictors of poor outcome and ICH, potentially affecting patients’ management in the ED.

According to our unpublished data, all 314 patients in the antiplatelet group have undergone a head computed tomography (HCT) during the first evaluation and 20/314 (6.36%) have had a first HCT positive for ICH. In total, 37/314 (11.8%) patients have been admitted to the Observation Unit and 1/37 (2.7%) has had a HCT positive for ICH after 24 h. The second HCT has been associated with trauma above the collarbone (OR 5.250, *p* 0.008), loss of consciousness (OR 4.111, *p* 0.007), and amnesia (OR 4.615, *p* 0.004). These clinical predictor factors have been associated with a second HCT but not with an intracranial hemorrhage (ICH). No differences have been shown for age, hemoglobin values, platelet counts, and PT-INR values between groups. On follow-up, no differences have been found between patients with or without repeated HCT. Our study demonstrates that APT is not a risk factor for ICH but it is related to a more severe clinical condition when bleeding does occur.

Thus, we strongly agree with the authors when they state that APT is not related to a higher risk of bleeding but rather related to a more severe clinical condition when bleeding does occur.

In our local protocol for MHT, we do not consider APT as a major risk factor for ICH. On one hand, patients with MHT and with low or green risk factors (no risk factors in pre-trauma and post-trauma) were discharged after clinical evaluation without a HCT scan and with specific instructions for head trauma. On the other hand, patients with MHT and one or more yellow risk factors are admitted into the Observation Unit for 6 h and a HCT scan is performed. Risk factors are divided in pre-trauma (more than 65 years old, epilepsy, alcohol or drugs abuse, neurosurgical intervention in the last 6 months, coagulopathy, or VKA or DOACs treatment) and post-trauma (loss of consciousness or trauma above the collarbone, worsening headache, vomit, or dangerous dynamic and post-trauma amnesia) factors. After the first HCT, if there is no ICH and no VKA or DOACs treatments, the patients are discharged with specific instructions for head trauma, while if there are VKA or DOACs treatments, the patients are observed for 24 h and usually a second CT scan is performed. However, according to our knowledge, there is a debate in the literature regarding the need for a second CT scan for this kind of patient if the clinical and neurological conditions remain stable.

We believe that, in line with other published data and authors, a second head CT scan in some conditions is useless, although to support this idea we need more data from a multicenter study in Italy.
